# Diversity of polyomaviruses and papillomaviruses in penguins from eastern and western Antarctica

**DOI:** 10.1099/mgen.0.001580

**Published:** 2025-11-24

**Authors:** Melanie Regney, Virginia Morandini, Annie E. Schmidt, Josabel Belliure, Amélie Lescroël, Megan Elrod, Amy Li, Aidan Cox, Danny Khor, Dennis Jongsomjit, Jean Pennycook, Christina Burnham, Nadia Swanson, Suzanne Winquist, Katie M. Dugger, David G. Ainley, Grant Ballard, Simona Kraberger, Arvind Varsani

**Affiliations:** 1 Biodesign Center for Fundamental and Applied Microbiomics, School of Life Sciences, Center for Evolution and Medicine, Arizona State University, Tempe, AZ 85287, USA; 2 Depto. Ecologia Evolutiva, Museo Nacional de Ciencias Naturales, CSIC, Madrid, Spain; 3 Point Blue Conservation Science, Petaluma, California, USA; 4 Global Change Ecology and Evolution Research Group (GloCEE), Department of Life Sciences, University of Alcalá, Madrid, Spain; 5 Oregon Cooperative Fish and Wildlife Research Unit, Department of Fisheries, Wildlife, and Conservation Sciences, Oregon State University, Hatfield Marine Science Center, Newport, OR 97365, USA; 6 U.S. Geological Survey, Oregon Cooperative Fish and Wildlife Research Unit, Department of Fisheries, Wildlife, and Conservation Sciences, Oregon State University, Corvallis, Oregon, USA; 7 H.T. Harvey & Associates Ecological Consultants, Los Gatos, California, USA; 8 Structural Biology Research Unit, Department of Integrative Biomedical Sciences, University of Cape Town, 7925 Cape Town, South Africa

**Keywords:** *Papillomaviridae*, *Polyomaviridae*, *Pygoscelis adeliae*, *Pygoscelis antarcticus*, *Pygoscelis papua*

## Abstract

Polyomaviruses and papillomaviruses are icosahedral viruses with small circular dsDNA genomes. Limited information on their diversity and evolution in avian hosts is available, with even less known regarding Antarctic penguins. Prior to this study, only one polyomavirus and two papillomaviruses had been identified in Adélie penguins (*Pygoscelis adeliae*). To expand our knowledge of these viruses in Antarctic penguins, we collected faecal and cloacal swab samples from 246 Adélie penguins over 3 breeding seasons (2021–2024) and 10 emperor penguins (*Aptenodytes forsteri*) during the 2023–2024 season on Ross Island (Ross Sea). Additionally, we sampled 66 Adélie, 40 chinstrap (*Pygoscelis antarcticus*) and 71 gentoo (*Pygoscelis papua*) penguins during the 2022–2023 season across various sites on the Antarctic Peninsula. All samples were screened for papillomaviruses and polyomaviruses. We identified 31 polyomaviruses in Adélie, gentoo and chinstrap penguins and 4 papillomaviruses in Adélie penguins sampled in both eastern and western Antarctica. The 31 penguin polyomaviruses belong to a single species but form four distinct variants that are host species specific with strong geographic clustering. The four papillomaviruses represent three different types, of which two are new types from Adélie penguins sampled on Yalour Island in the West Antarctic Peninsula. Co-occurrence of two polyomavirus variants was identified in two individual gentoo penguins. Both of these variants appear to be circulating in gentoo penguins at Cierva Cove, Hope Bay in Trinity Peninsula along the Antarctic Peninsula, and at Hannah Point on Livingstone Island and Stranger Point on King George Island in the South Shetland Islands. Here, we expand the known diversity, host and geographical ranges of penguin polyomaviruses and, together with a previously identified polyomavirus on Ross Island from 2012 to 2013, show that they form five distinct lineages. The four papillomaviruses identified in this study, together with two previously identified from Ross Island in 2012 and 2013 breeding seasons, show substantial diversity reflecting four papillomavirus types across three viral species and two distinct genera. Continued surveillance and viral genomic analysis across a larger geographical framework will help understand the evolution, transmission and incidence rates of these viruses.

**Impact Statement**

There is little known about the viruses circulating amongst animals that breed in Antarctica in comparison to other regions in the world. Prior to this study, two papillomaviruses and one polyomavirus had been identified in Adélie penguins sampled on Ross Island in western Antarctic. In this study, we expand the limited knowledge of papillomaviruses and polyomaviruses circulating amongst three Antarctic penguins, Adélie penguins (*Pygoscelis adeliae*), chinstrap (*Pygoscelis antarcticus*) and gentoo penguins (*Pygoscelis papua*) breeding across various sites in eastern and western Antarctica. We sampled and screened 433 penguin adult and chick samples (303 cloacal swabs and 130 faecal samples) across 3 breeding seasons. We identified 31 polyomaviruses in adult Adélie (*n*=7), gentoo (*n*=22) and chinstrap (*n*=2) penguins in western and eastern Antarctica and show that these 31 penguin polyomaviruses belong to a single species but form 4 distinct variants that are host species specific with strong geographic clustering. For the first time, we detected co-occurrence of two polyomavirus variants in two individual penguins. Additionally, we identified four papillomaviruses in adult Adélie penguins; two are new papillomavirus types from Adélie penguins sampled from the West Antarctic Peninsula. Although the study did not identify any disease amongst the penguins sampled, it does highlight that there is diversity within these viruses with some host and location specificity within virus lineages.

## Data Summary

The polyomavirus genomes have been deposited in GenBank under accession numbers PQ583410–PQ583440 and the papillomaviruses under PQ583406-PQ583409. Raw sequence data have been deposited under BioProject # PRJNA874327, BioSample # SAMN44562079–SAMN44562112 and SRA #s SRR31251300–SRR31251333.

## Introduction

Penguins (Aves: Sphenisciformes) are arguably Antarctica’s most iconic animals [1]. Amongst the five penguin species that breed along the Antarctic coastline and nearby islands are the high-latitude emperor (*A. forsteri*) and Adélie penguins (*Pygoscelis adeliae*) and at lower latitudes, the chinstrap (*Pygoscelis antarcticus*), gentoo (*Pygoscelis papua*) and macaroni penguins (*Eudyptes chrysolophus*) [2,3].

**Fig. 1.**
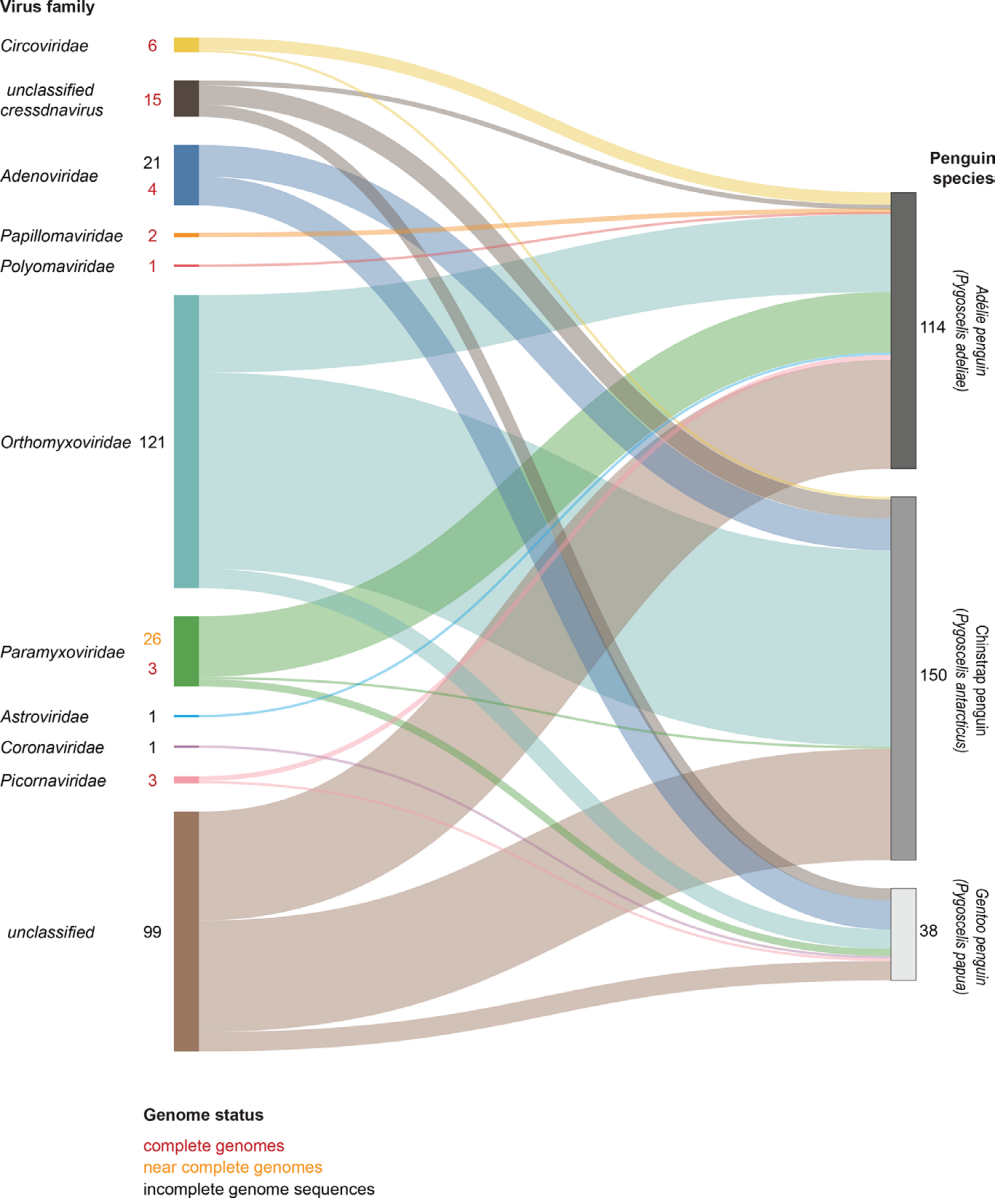
Summary of the virus sequences with complete, near-complete (coding complete) or incomplete genome sequences (partial) identified in Antarctic penguins based on sequence data available in the NCBI Virus [24] prior to this study. The Sankey plot was generated using SankeyMATIC (https://github.com/nowthis/sankeymatic) and shows the number of sequences of viruses in various families and their source/host.

Despite extensive biological and ecological research on penguins [2,3], little is known about viruses that infect them [4]. To date, ~302 viral genomic sequences have been identified in association with Antarctic penguins. These include adenoviruses [5,6], astroviruses [7,8], paramyxoviruses [8,11], coronaviruses [8], circoviruses [12,13], orthomyxoviruses [8,14,16], papillomaviruses [17,18], picornaviruses [19,21], a polyomavirus [22] and a suite of unclassified viruses [8,23]. A summary of the virus sequences available in the National Center for Biotechnology Information (NCBI) Virus [24] found associated with various penguin species south of the polar front is provided in Fig. 1. Of these, complete genomes are available only for adenoviruses (*n*=4), circoviruses (*n*=6), papillomaviruses (*n*=2), paramyxoviruses (*n*=3), picornaviruses (*n*=3) and a polyomavirus (*n*=1). Twenty-six genomes of paramyxoviruses are nearly complete >14,500 nt (Fig. 1).

Of the many avian-associated viruses, mardiviruses, papillomaviruses and retroviruses are oncogenic [25,26]. Polyomaviruses and papillomaviruses are classified into the orders *Sepolyvirales* and *Zurhausenvirales*, respectively, both within class *Papovaviricetes* of phylum *Cossaviricota* [27,28]. They share some similarities in their Super Family 3 helicase domain, single jelly roll capsid protein [29], suggesting a shared evolutionary origin. They also have analogous mechanisms of replication and host cell-cycle modulation and, in some cases, the ability to induce tumours [25,26]. Both polyomaviruses and papillomaviruses appear to be relatively ubiquitous across vertebrates with some evidence of coevolution/co-speciation with host lineages [25,30,36]. In general, both polyomaviruses (~10^−5^–10^−7^ subs/per/site/year) [30,36,38] and papillomaviruses (~10^−7^–10^−8^ subs/per/site/year) [32,33, 39,43] have relatively low substitution rates compared to other viruses [44].

Polyomaviruses (family *Polyomaviridae*) have small, circular dsDNA genomes (3.9 to 7.4 kb) encapsidated into icosahedral capsids [27,45, 46]. Polyomaviruses infect a wide variety of hosts, including mammals [31,47,49], birds [50,51], fish [30,46, 52,54] and arthropods [30,55]. Currently, polyomaviruses are classified into 118 species assigned to eight genera: *Alphapolyomavirus*, *Betapolyomavirus*, *Deltapolyomavirus*, *Epsilonpolyomavirus*, *Etapolyomavirus*, *Gammapolyomavirus*, *Thetapolyomavirus* and *Zetapolyomavirus* [27]. The currently known avian polyomaviruses are classified in the genus *Gammapolyomavirus*. Some avian polyomaviruses have been linked to severe diseases in birds, such as budgerigar fledgling disease affecting the host’s immune system and functions and goose haemorrhagic polyomavirus leading to haemorrhagic nephritis and enteritis in domestic geese [50,51, 56,61].

Papillomaviruses have circular dsDNA genomes (~5.7 to 8.6 kb) [28,53, 62] that are encapsidated into icosahedral capsids [63]. Papillomaviruses are classified in the family *Papillomaviridae* under two subfamilies, *Firstpapillomavirinae* and *Secondpapillomavirinae* [28]. These viruses are generally host- and tissue-specific within the host’s epithelial cells, such as skin, squamous and mucosal cells of mammalian, avian, reptilian and fish hosts. There are ~730 papillomavirus types listed on the Papillomavirus Episteme (https://pave.niaid.nih.gov) [64]. Of these, many have been classified in 53 genera, though many, especially those associated with non-mammalian (e.g. avian) hosts, remain unclassified [28]. The range of known host species is broad, mainly comprising humans and other mammals, but also includes birds, reptiles and fish [62,64].

In the context of Antarctic animals, polyomaviruses have been identified in two ice fish species (emerald notothen, *Trematomus bernacchii*; sharp-spined notothen, *Trematomus pennellii*) [30,54], a Weddell seal (*Leptonychotes weddellii*) [49] and Adélie penguins [22], all from Ross Island/Ross Sea. On the other hand, papillomaviruses have been previously identified in Weddell seals [65,66], an Emerald notothen (*T. bernacchii*) [62] and two Adélie penguins [17,18] in the Ross Island/Ross Sea region and Antarctic fur seals (*Arctocephalus gazella*) and leopard seals (*Hydrurga leptonyx*) [65] in the Antarctic Peninsula.

Through collaborative efforts, this study expands on previous work done by our team on penguin polyomavirus [22] and papillomaviruses [17,18] to investigate the incidence and viral diversity of polyomaviruses and papillomaviruses in Adélie, emperor, chinstrap and gentoo penguins across eastern and western Antarctica. We focus on polyomaviruses and papillomaviruses for this study because these viruses have some level of host lineage specificity. Therefore, collecting spatial and temporal datasets of polyomaviruses and papillomaviruses associated with Antarctic penguins can enhance our understanding of (1) virus–host interactions and viral evolution in these flightless birds compared to flying birds, (2) geographical movement/restriction of penguin species/populations by proxy of viral lineages, e.g. JC polyomavirus and human dispersal [37,67], and (3) animal health in a rapidly changing Antarctic ecosystem as a consequence of climate change. Here, our results expand on the limited knowledge of polyomaviruses and papillomaviruses associated with Antarctic penguins and contribute towards assembling these spatial and temporal datasets across multiple penguin species and provide insights into the diversity of these viruses.

## Methods

### Sample collection and storage

Faecal and cloacal swab samples of chick and adult Adélie penguins over three breeding seasons (2021–2023) and faecal samples from adult emperor penguins during the 2023 season were collected from breeding colonies on Ross Island in eastern Antarctica (Table 1). The samples were collected under the Antarctic Conservation Act Permit from NSF through Point Blue Conservation Science with logistics provided by the U.S. Antarctic Program NSF grant ANT 1935870 and 2040199. From the Antarctic peninsula region, cloacal swabs from adult Adélie penguins, chinstrap penguins and gentoo penguins were collected over the 2022 breeding season under permit # CPE‐2021‐8 with logistic support from Comité Polar Español, Ministerio de Ciencia, Innovación y Universidades. All samples were collected under Institutional Animal Care and Use Committee (IACUC) approved protocols of Point Blue Conservation Science and Museo Nacional de Ciencias Naturales.

**Table 1.** Summary of the sample numbers, penguin species and sampling location

Region	Location GPS coordinate	Breeding season	Penguin species	Cloacal swab	Faecal sample	Total sample
Ross Island	Cape Crozier, Ross Island −77.452523, 169.231781	2021	Adélie penguin	Chicks: 23	Adult: 10 Chicks: 26	59
Ross Island	Cape Crozier, Ross Island −77.452523, 169.231781	2022	Adélie penguin	Adult: 59	Adult: 28 Chicks: 13	100
Ross Island	Cape Crozier, Ross Island −77.452523, 169.231781	2023	Adélie penguin	Adult: 16	Adult: 14 Chicks: 8	38
Ross Island	Cape Royds, Ross Island −77.554615, 166.161374	2022	Adélie penguin	Adult: 9	Adult: 3 Chicks: 6	18
Ross Island	Cape Royds, Ross Island −77.554615, 166.161374	2023	Adélie penguin	Adult: 9	Adult: 15 Chicks: 7	31
Ross Island	Cape Crozier, Ross Island −77.457495, 169.294532	2023	Emperor penguin	–	Adult: 10	10
Antarctic Peninsula	Avian Island –67.770876, –68.886246	2022	Adélie penguin	Adult: 25	–	25
Antarctic Peninsula	Hope Bay, Trinity Peninsula –63.383333, –56.983333	2022	Adélie penguin	Adult: 6	–	6
Antarctic Peninsula	Stranger Point, King George Island, South Shetland Islands –62.26175, –58.61756	2022	Adélie penguin	Adult: 10	–	10
Antarctic Peninsula	Yalour Island –65.233333, –64.166667	2022	Adélie penguin	Adult: 25	–	25
Antarctic Peninsula	Hannah Point, Livingston Island, South Shetland Islands –62.65444, –60.61342	2022	Chinstrap penguin	Adult: 15	–	15
Antarctic Peninsula	Penguin Island, South Shetland Islands –62.1, –57.928056	2022	Chinstrap penguin	Adult: 10	–	10
Antarctic Peninsula	Georges Point, Ronge Island –64.666667, –62.666667	2022	Chinstrap penguin	Adult: 15	–	15
Antarctic Peninsula	Cierva Cove –64.155766, –60.955183	2022	Gentoo penguin	Adult: 21	–	21
Antarctic Peninsula	Hannah Point, Livingston Island, South Shetland Islands –62.65444, –60.61342	2022	Gentoo penguin	Adult: 15	–	15
Antarctic Peninsula	Hope Bay, Trinity Peninsula –63.383333, –56.983333	2022	Gentoo penguin	Adult: 6	–	6
Antarctic Peninsula	Stranger Point, King George Island, South Shetland Islands –62.26175, –58.61756	2022	Gentoo penguin	Adult: 16	–	16
Antarctic Peninsula	Hannah Point, Livingston Island, South Shetland Islands –62.65444, –60.61342	2022	Gentoo penguin	Adult: 13	–	13

For this study, we accessed aliquots of samples collected for other studies, and thus, we did not target specific animals with signs of disease. Nonetheless, for all animals from which samples were taken, no visible pathology, lesions or warts were observed at the time of sampling (Table 1). Faecal samples were collected in 50 ml conical tubes and cloacal swabs were stored in Universal Transport Medium (UTM; Copan Diagnostics, USA). All samples were stored and shipped to the Varsani Lab at Arizona State University, USA, at −20 °C under USDA-APHIS permit # 639-23-90-86196, where they were stored at −80 °C until processing.

### Viral DNA extraction and circular DNA enrichment

Approximately 2 g of each faecal sample was resuspended in 2 ml of sodium chloride and magnesium sulphate (SM) buffer [0.1 M NaCl, 50 mM Tris-HCl (pH 7.4), 8 mM MgSO_4_, and 0.01% gelatin) (G-Biosciences, USA) by vortexing. The homogenized matter was centrifuged at 10,000 x ***g*** for 20 min, and the resulting supernatant was sequentially filtered through 0.45 and 0.22 µm pore-sized syringe filters. For each cloacal swab sample, 1 ml of the UTM viral transport media was filtered through a 0.22 µm pore-sized syringe filter. From the resulting filtrate, 200 µl was taken to extract viral DNA using the High Pure viral Nucleic Acid Kit (Roche Diagnostics, USA) following the manufacturer’s instructions. Rolling circle amplification (RCA) was performed to enrich the circular DNA molecules in viral DNA purification using the Templiphi 100 Amplification Kit with DNA Polymerase Phi29 (Cytiva Lifesciences, USA).

### High-throughput sequencing and *de novo* assemblies

For each sample, an equal ratio of purified viral DNA together with its respective RCA product was used to generate Illumina sequencing libraries using the Illumina DNA library preparation (M) tagmentation kit (Illumina, USA) and sequenced on an Illumina NovaSeq X Plus sequencer (Illumina) at Psomagen Inc. (USA).

The Illumina sequence raw reads were trimmed using Trimmomatic v0.39 [68], and then, the paired-end reads were *de novo* assembled using MEGAHIT v1.2.9 [69]. The assembled contigs (>1,000 nt) were analysed for similarity to known protein sequences using DIAMOND blastx [70] against a viral RefSeq database (release 220). Contigs were determined to be circular based on terminal redundancy.

Translation products of 30 contigs were found to have high similarities to proteins encoded by polyomaviruses, and 29 of these represented circular complete genomes. In four individual penguin samples, four *de novo* assembled circular contigs with high similarity to papillomavirus sequences were identified. ORFs in all genomes were determined using ORFinder and manually edited to account for spliced coding regions for polyomavirus large and small tumour antigens (T-antigen), in Geneious Prime v2022.0.2 (Dotmatics, USA).

### Recovery of complete viral genomes and Sanger sequencing

CoverM [71] was used to map raw reads from each sample to the assembled genomes to identify any samples with poor genome coverage for targeted PCR-based amplification of the full genome. To determine the complete genomes of the two partial polyomavirus sequences, primers described in [22] were utilized with KAPA HiFi HotStart ReadyMix (Roche Diagnostics) using the following thermal cycling protocol: initial denaturation at 95 °C for 3 min followed by 25 cycles of 98 °C for 20 s, 60 °C for 15 s and 72 °C for 5 min and a final extension at 72 °C for 5 min. The resulting amplicons were resolved on a 0.7% agarose gel (stained with SYBR Safe DNA gel stain). Amplicons of ~5 kb were gel-excised and purified using MEGAquick-spin plus (iNTRON, South Korea). The purified products were ligated into the plasmid pJET1.2 (Thermo Fisher Scientific, USA) and transformed into XL1-blue competent *Escherichia coli* cells. The recombinant plasmids were purified using the Fast DNA-spin (iNTRON) and Sanger-sequenced by primer walking at Macrogen Inc. (South Korea). The Sanger sequence reads were trimmed and assembled using Geneious Prime v2022.0.2 (Dotmatics).

### Polyomavirus sequence analyses

We generated a dataset for the polyomavirus analysis that included the 31 penguin polyomaviruses identified in this study together with representative sequences in the genera *Gammapolyomavirus* and *Thetapolyomavirus* (as an outgroup). From these, the large T-antigen, small T-antigen, VP1 and VP2 coding sequences were extracted and translated.

All nucleotide and amino acid pairwise identities were determined using SDT v1.2 [72]. Amino acid sequence logos of conserved motifs and domains in the large T-antigen protein were generated using WebLogo3 [73].

The translated sequences of the large T-antigen were aligned using MAFFT 7 [74]. The alignment was used to infer a maximum-likelihood phylogenetic tree using IQ-TREE 2 [75] with LG+I+G4 identified as the best-fit model using ModelFinder [76]. Branches with 80% approximate likelihood-ratio test (aLRT) support were collapsed using TreeGraph2 [77], and the phylogenetic tree was rooted with sequences of the representative members of *Thetapolyomavirus*.

### Papillomavirus sequence analyses

A dataset of avian and reptilian (as an outgroup) papillomaviruses was generated from PAVE [64] and combined with the four papillomaviruses identified in this study. From these, the E1, E2, E6, E7 and E9 and L1 and L2 coding regions were extracted and translated.

All nucleotide and amino acid pairwise identities were determined using SDT v1.2 [72]. Amino acid sequence logos of conserved motifs and domains in the E6 and E7 proteins were generated using WebLogo3 [73].

For phylogenetic analysis, the E1, E2 and L1 amino acid sequences were aligned using MAFFT 7 [74]. The alignments were trimmed using TrimAL [78] and then concatenated (E1+E2+L1). The concatenated alignment was used to infer a maximum likelihood phylogenetic tree utilizing IQ-TREE 2 [75] in partition mode with models LG+I+G, LG+I+G+F and LG+I+G+F which were determined to be the best fit substitution models for E1, E2 and L1, respectively, by ProtTest 3 [79]. Branches with 80% aLRT support were collapsed using TreeGraph2 [77], and the phylogenetic tree was rooted with *Hemidactylus frenatus* papillomavirus 1 (HfrePV1) and *Hemidactylus frenatus* papillomavirus 2 (HfrePV2) (MK207055, MN194600) [80].

### Recombination analysis

For recombination analysis, we aligned the genome datasets of avian polyomaviruses and papillomaviruses individually using MAFFT 7 [74]. We used these alignments to detect recombination using RDP5 [81] with default settings. Recombination signals with an associated *P*-value <0.05 and detected by three or more recombination methods implemented in RDP5, coupled with strong phylogenetic support, were accepted as plausible evidence of recombination.

## Results and discussion

**Fig. 2.**
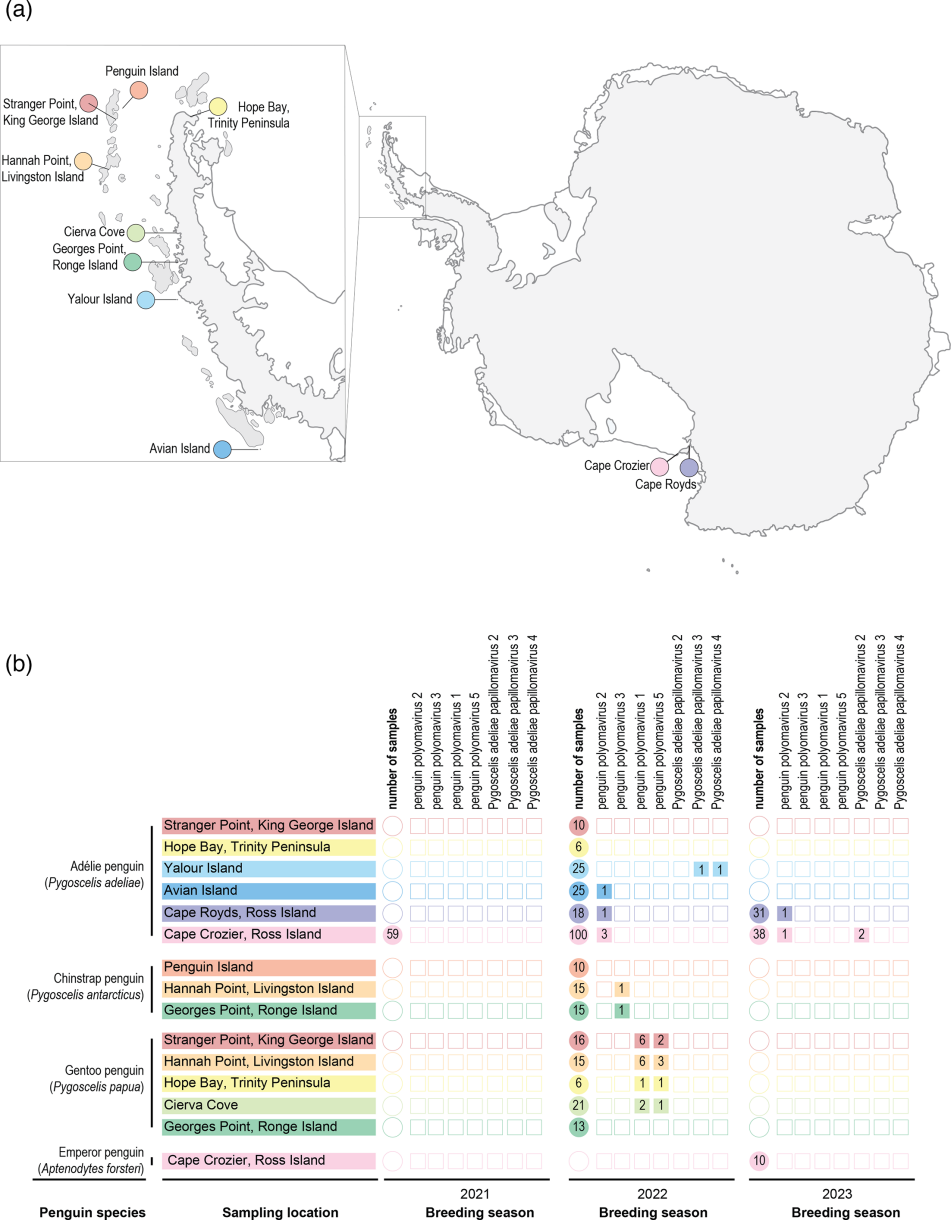
(a) Sampling regions in western and eastern Antarctica with inset maps of the Antarctic Peninsula and Ross Island. Sampling sites on the Antarctic Peninsula include Avian Island, Cierva Cove and Georges Point on Ronge Island; Hope Bay on Trinity Peninsula, Yalour Island along the Antarctic Peninsula and Hannah Point on Livingston Island; and Penguin Island and Stranger Point on King George Island in South Shetland Islands. On Ross Island, sampling locations include Cape Royds and Cape Crozier. (b) Summary of the polyomaviruses and papillomaviruses identified across the ten Antarctic sampling locations during the three breeding/field seasons. The sampling locations are colour-coded and listed by penguin species. Filled circles with numbers depict the sample number and filled squares with numbers denote positive samples for the polyomavirus and papillomavirus types.

In the *de novo* assembled contigs, we identified various bacteriophages (primarily those in the phyla *Phixviricota* and *Uroviricota*) and eukaryote-associated viruses (in the phyla *Cossaviricota*, *Cressdnaviricota* and *Preplasmiviricota*). For the purpose of this study, we focus on cossaviricots (polyomaviruses and papillomaviruses) that are known to infect vertebrate hosts. We screened the viral-like *de novo* assembled contigs for polyomaviruses and papillomaviruses from a total of 433 samples, with 256 of the samples being from Adélie penguins (*n*=246) spanning three breeding seasons (2021, 2022 and 2023) and emperor penguins (*n*=10) from the 2023 breeding season on Ross Island. Another 177 samples (Adélie penguin, *n*=66; chinstrap penguin, *n*=40; and gentoo penguin, *n*=71) were collected during the 2022 breeding season across various sites along the Antarctic Peninsula. We identified a total of 31 polyomavirus and 4 papillomavirus genomes across all these samples (Table 2, Fig. 2). These genomes were cross-checked with the raw reads from all the samples, and we did not identify any additional polyomavirus and papillomavirus sequences. No polyomaviruses or papillomaviruses were identified in the 59 samples of Adélie penguins from Cape Crozier during the 2021 breeding season, and none were identified in the 10 emperor penguin faecal samples from the Cape Crozier sampled in the 2023 breeding season. Our sample set included 83 Adélie penguin chicks from Ross Island (2021, 2022 and 2023 breeding seasons). None of these were positive for polyomaviruses or papillomaviruses, i.e. only adults were found to be positive. We did not find any co-occurrence in any individual of polyomavirus and papillomaviruses. We did, however, find two individual gentoo penguins (sampled at Hannah Point on Livingston Island in the South Shetland Islands) with co-occurrence with two different polyomavirus lineages. We did not find any evidence of recombination in the polyomavirus and papillomavirus genomes.

**Table 2.** Summary of the polyomaviruses and papillomaviruses (with GenBank accession numbers) identified from Adélie penguins, chinstrap penguins and gentoo penguins from Ross Island (eastern Antarctica) and the Antarctic Peninsula (western Antarctica)

Accession #	Virus	Sample ID	Sampling date	Location	Penguin species	Sample type
PQ583410	Penguin polyomavirus 2	PyV_c_2223_c09	16 December 2022	Cape Crozier, Ross Island	Adélie penguin	Cloacal swab
PQ583411	Penguin polyomavirus 2	PyV_c_2223_c48	31 December 2022	Cape Crozier, Ross Island	Adélie penguin	Cloacal swab
PQ583412	Penguin polyomavirus 2	PyV_c_2223_c55	7 January 2023	Cape Crozier, Ross Island	Adélie penguin	Cloacal swab
PQ583413	Penguin polyomavirus 2	PyV_c_2324_c10	21 December 2023	Cape Crozier, Ross Island	Adélie penguin	Cloacal swab
PQ583417	Penguin polyomavirus 2	PyV_PAD_23_33	23 January 2023	Avian Island	Adélie penguin	Cloacal swab
PQ583414	Penguin polyomavirus 2	PyV_f_2324_r19	3 January 2024	Cape Royds, Ross Island	Adélie penguin	Faeces
PQ583440	Penguin polyomavirus 2	PyV_c_2223_r21	10 January 2023	Cape Royds, Ross Island	Adélie penguin	Cloacal swab
PQ583415	Penguin polyomavirus 3	PyV_PA_23_12	8 January 2023	Hannah Point, Livingston Island	Chinstrap penguin	Cloacal swab
PQ583416	Penguin polyomavirus 3	PyV_PA_23_37	18 January 2023	Georges Point, Ronge Island	Chinstrap penguin	Cloacal swab
PQ583419	Penguin polyomavirus 4	PyV_PP_23_04	1 January 2023	Hannah Point, Livingston Island	Gentoo penguin	Cloacal swab
PQ583420	Penguin polyomavirus 4	PyV_PP_23_05	1 January 2023	Hannah Point, Livingston Island	Gentoo penguin	Cloacal swab
PQ583421	Penguin polyomavirus 4	PyV_PP_23_06b	1 January 2023	Hannah Point, Livingston Island	Gentoo penguin	Cloacal swab
PQ583423	Penguin polyomavirus 4	PyV_PP_23_09 a	1 January 2023	Hannah Point, Livingston Island	Gentoo penguin	Cloacal swab
PQ583425	Penguin polyomavirus 4	PyV_PP_23_11	7 January 2023	Hannah Point, Livingston Island	Gentoo penguin	Cloacal swab
PQ583426	Penguin polyomavirus 4	PyV_PP_23_14	7 January 2023	Hannah Point, Livingston Island	Gentoo penguin	Cloacal swab
PQ583427	Penguin polyomavirus 4	PyV_PP_23_20	10 January 2023	Cierva Cove	Gentoo penguin	Cloacal swab
PQ583429	Penguin polyomavirus 4	PyV_PP_23_28	18 January 2023	Stranger Point, King George Island	Gentoo penguin	Cloacal swab
PQ583432	Penguin polyomavirus 4	PyV_PP_23_36	18 January 2023	Stranger Point, King George Island	Gentoo penguin	Cloacal swab
PQ583433	Penguin polyomavirus 4	PyV_PP_23_37	18 January 2023	Stranger Point, King George Island	Gentoo penguin	Cloacal swab
PQ583434	Penguin polyomavirus 4	PyV_PP_23_40	18 January 2023	Stranger Point, King George Island	Gentoo penguin	Cloacal swab
PQ583436	Penguin polyomavirus 4	PyV_PP_23_44	16 January 2023	Hope Bay, Trinity Peninsula	Gentoo penguin	Cloacal swab
PQ583437	Penguin polyomavirus 4	PyV_PP_23_50	18 January 2023	Stranger Point, King George Island	Gentoo penguin	Cloacal swab
PQ583438	Penguin polyomavirus 4	PyV_PP_23_54	18 January 2023	Stranger Point, King George Island	Gentoo penguin	Cloacal swab
PQ583439	Penguin polyomavirus 4	PyV_PP_23_71	29 January 2023	Cierva Cove	Gentoo penguin	Cloacal swab
PQ583418	Penguin polyomavirus 5	PyV_PP_23_02	1 January 2023	Hannah Point, Livingston Island	Gentoo penguin	Cloacal swab
PQ583422	Penguin polyomavirus 5	PyV_PP_23_06 a	1 January 2023	Hannah Point, Livingston Island	Gentoo penguin	Cloacal swab
PQ583424	Penguin polyomavirus 5	PyV_PP_23_09b	1 January 2023	Hannah Point, Livingston Island	Gentoo penguin	Cloacal swab
PQ583428	Penguin polyomavirus 5	PyV_PP_23_22	10 January 2023	Cierva Cove	Gentoo penguin	Cloacal swab
PQ583430	Penguin polyomavirus 5	PyV_PP_23_34	18 January 2023	Stranger Point, King George Island	Gentoo penguin	Cloacal swab
PQ583431	Penguin polyomavirus 5	PyV_PP_23_35	18 January 2023	Stranger Point, King George Island	Gentoo penguin	Cloacal swab
PQ583435	Penguin polyomavirus 5	PyV_PP_23_41	16 January 2023	Hope Bay, Trinity Peninsula	Gentoo penguin	Cloacal swab
PQ583406	Pygoscelis adeliae papillomavirus 2	PV_c_2324_c05	18 December 2023	Cape Crozier, Ross Island	Adélie penguin	Cloacal swab
PQ583407	Pygoscelis adeliae papillomavirus 2	PV_c_2324_c06	19 December 2023	Cape Crozier, Ross Island	Adélie penguin	Cloacal swab
PQ583409	Pygoscelis adeliae papillomavirus 3	PV_PAD_23_63	28 January 2023	Yalour Island	Adélie penguin	Cloacal swab
PQ583408	Pygoscelis adeliae papillomavirus 4	PV_PAD_23_57	28 January 2023	Yalour Island	Adélie penguin	Cloacal swab

### Penguin polyomaviruses

Considering the 350 adult and 83 chick penguin samples, we identified 31 polyomaviruses from cloacal swabs and 1 faecal sample collected from 7 Adélie, 2 chinstrap and 22 gentoo penguins (Fig. 2). Prior to this study, the only known polyomavirus in penguins was Adélie penguin polyomavirus 1, classified as *Gammapolyomavirus padeliae* species, from an Adélie penguin sampled at Cape Royds (2012 breeding season) on Ross Island [22], eastern Antarctica. As part of this study, we identified six polyomaviruses in Adélie penguins sampled in eastern Antarctica: one each in 2022 and 2023 breeding seasons at Cape Royds and three in 2022 and one in 2023 breeding season at Cape Crozier (Table 2, Fig. 2). In western Antarctica in the 2022 breeding season, we identified polyomaviruses from an Adélie penguin at Avian Island, one each from chinstrap penguins at Georges Point on Ronge Island and Hannah Point on Livingston Island, and a total of 22 from gentoo penguins, 3 at Cierva Cove, 2 at Hope Bay in the Trinity Peninsula, 9 at Hannah Point on Livingston Island and 8 at Stranger Point on King George Island.

The genome organization of the 31 polyomavirus genomes is similar to that of other avian polyomaviruses, with their genome sizes ranging from 4,940 to 4,987 nt with an average G+C content of 52.9–53.4 mol%. In brief, their genomes have bidirectionally organized coding regions for early and late transcription separated by a non-coding control region which has the origin of viral DNA replication [27,82]. The early transcribed genes encode the large and small tumour antigens (T-antigen), essential for viral replication and oncogenesis, where the late transcribed genes encode three structural proteins (VP1, VP2 and VP3) that assemble into icosahedral viral capsid [27,82,84].

**Fig. 3.**
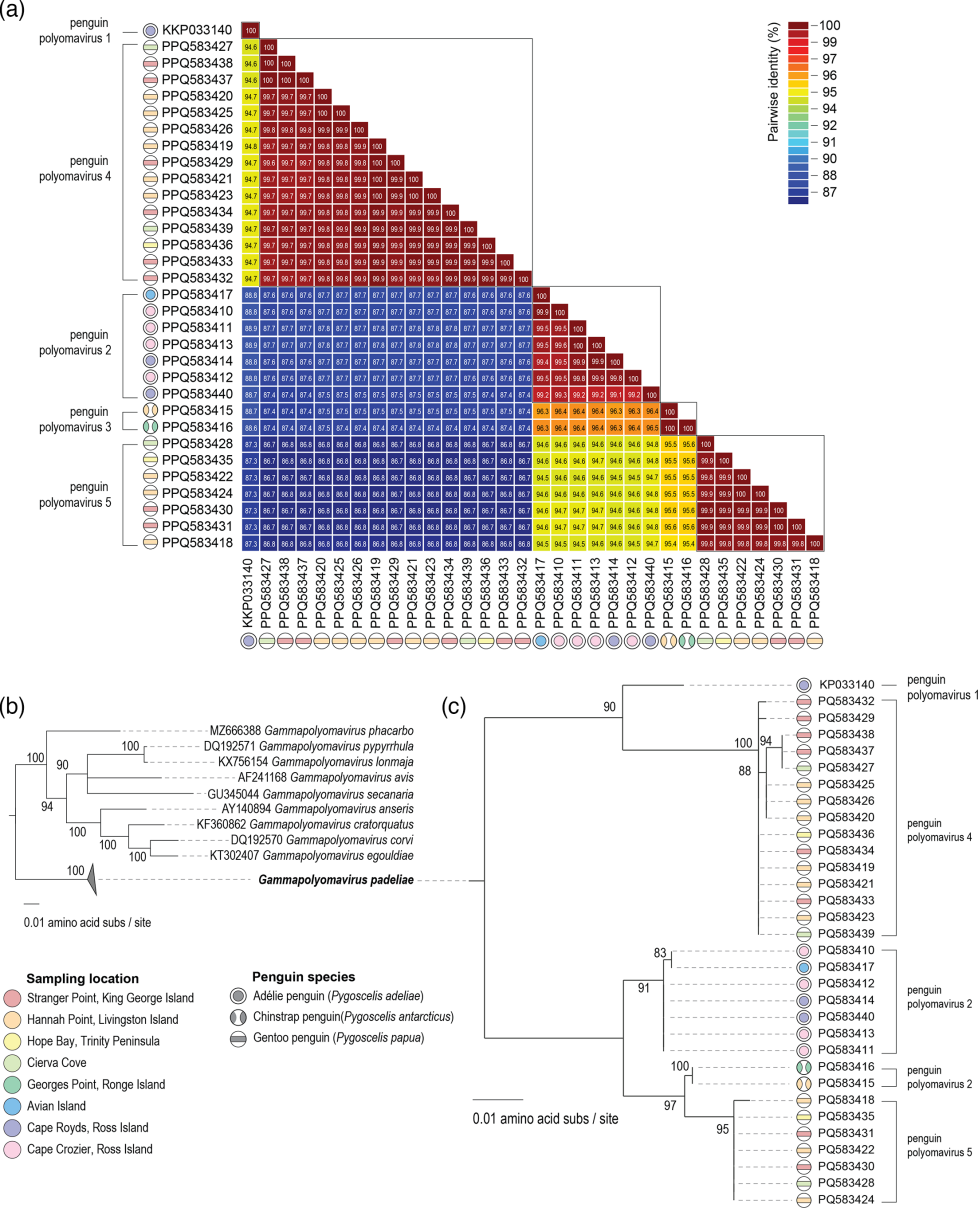
(a) Genome-wide pairwise identity matrix of the polyomavirus sequences identified in this study and the one previously identified from an Adélie penguin in the 2012 breeding season by Varsani et al. [22]. (b) A maximum-likelihood phylogenetic tree of large T-antigen protein sequences of representative members of the *Gammapolyomavirus* genus. (c) Maximum-likelihood phylogenetic tree of the members of the species *Gammapolyomavirus padeliae*. Branches with <80% branch support have been collapsed using TreeGraph2 [77].

The International Committee on Taxonomy of Viruses (ICTV) *Polyomaviridae* study group has established an 85% amino acid identity of the large T-antigen protein sequence as a species demarcation threshold [27]. The large T-antigen of the 31 polyomaviruses identified in this study, together with the one identified by [22], shares >91% identity (Fig. S1, available in the online Supplementary Material) and thus all belong to the species *Gammapolyomavirus padeliae*. The 31 polyomaviruses share >86% genome-wide pairwise identity. They form four variant groupings that are host species-specific lineages and members of each lineage share >99% genome-wide identity with each other and <97% with members of any of the other four lineages (Fig. 3). These five variant groupings are supported by the maximum-likelihood phylogenetic tree of the large T-antigen protein sequences of the viruses in the species *Gammapolyomavirus padeliae* (Fig. 3). The penguin polyomavirus 1 from Cape Royds in the 2012 breeding season [22] is a singleton representing a unique variant. The six polyomaviruses from Ross Island Adélie penguins from this study, together with one from Avian Island (Antarctic Peninsula), are members of the second variant penguin polyomavirus 2 (Fig. 3). The two polyomaviruses from chinstrap penguins from Georges Point on Ronge Island and Hannah Point on Livingston Island represent the third lineage, penguin polyomavirus 3. The polyomaviruses from gentoo penguins form two lineages: penguin polyomavirus 4 (*n*=15) and penguin polyomavirus 5 (*n*=7). Both lineages appear to be present in the sampling locations at Cierva Cove and Hope Bay on Trinity Peninsula and Hannah Point on Livingston Island and Stranger Point on King George in the South Shetland Islands.

There is a clear lineage structure based on penguin species and to some extent geographical location of breeding colonies (eastern or western Antarctica). It is important to note that two of the gentoo penguins (PP_23_06 and PP_23_09) from Hannah Point on Livingston Island (Table 2, Fig. 3) were hosts to two lineages of polyomaviruses: penguin polyomavirus 4 (PQ583421 and PQ583423) and penguin polyomavirus 5 (PQ583422 and PQ583424). This is evidence of co-occurrence with the two polyomavirus variants that are circulating amongst the gentoo penguins at Cierva Cove, Hope Bay in Trinity Peninsula and Hannah Point on Livingston Island and Stranger Point on King George Island in the South Shetland Islands. It is worth noting that of the 71 gentoo penguin samples collected in the 2022 breeding season across 5 locations in the Antarctic Peninsula region, we have detected 18 individuals that have either penguin polyomavirus 3 or penguin polyomavirus 4, with 2 having a co-occurrence. These two polyomaviruses have high incidence with ~50% positives at Hannah Point on Livingston Island (7 out of 15 individuals) and Stranger Point on King George Island (8 out of 16 individuals) probably indicating environmental niche overlap amongst colonies in the South Shetland Islands [85]. At Georges Point on Ronge Island, none of the gentoo penguins sampled had either penguin polyomavirus 4 or 5 variants, and only 1 chinstrap penguin out of 15 was positive for penguin polyomavirus 3. In general, the overall incidence of polyomavirus 4 was 21% and 10% for penguin polyomavirus 5, with these variants contributing to a 31% overall incidence rate amongst gentoo penguins in the Antarctic Peninsula. In contrast, the incidence of penguin polyomavirus 3 was 5% in chinstrap penguins, while penguin polyomavirus 2 had an incidence of 4% in Adélie penguins sampled in the Antarctic Peninsula and 1.6% in those from Ross Island. Avian polyomavirus studies have mainly focused on the following: (1) budgerigar fledgling disease virus [50,86, 87], with reported prevalence ranging from 0.8% to 25% in psittacine species, although many of these are captive populations [88,94], and (2) goose haemorrhagic polyomavirus in domestic ducks and geese with prevalence ranging from 10% to 59% [61,95, 96].

Although studies on avian polyomaviruses (in particular those in the species *Gammapolyomavirus avis*) have hypothesized that incidence rates are higher in juvenile birds, manifesting in more severe disease outcomes compared to adults [97], we did not detect any polyomavirus in Adélie penguin chicks. This finding (or lack of a finding), along with the lack of detection in the 10 emperor penguin samples, may reflect methodological limitations (opportunistic faeces collection vs. cloacal swabs) or a substantially lower sample size in the case of emperor penguins and chicks.

A more global analysis shows that the five lineages of penguin polyomaviruses share 58.9–62.4% genome-wide pairwise identity with other avian polyomaviruses (Fig. S1 in Supplementary Material). The protein sequences encoded by the penguin polyomaviruses share 47–53% amino acid identity for large T-antigen, 34–47% for the small T-antigen, 55–59% for the VP1 and 37–42% for VP2 with those of other avian polyomaviruses (Fig. S1 in Supplementary Material).

Within the large T-antigen protein sequences, we identified the conserved motifs – conserved region 1 (CR1; LIRLL), the hexapeptide (DnaJ; HPDKGG), the retinoblastoma protein binding motif (pRB; LYCEE), the putative nuclear localization signal (NLS; PPKSQP), the zinc-finger motif (CQDCQQQRANTPVGKLKRKWIGGHCDDH) and the ATPase motifs (GPVNSKT and GSVPVNLE) [98] which are summarized in Fig. S2 (Supplementary Material).

### Penguin papillomaviruses

**Fig. 4.**
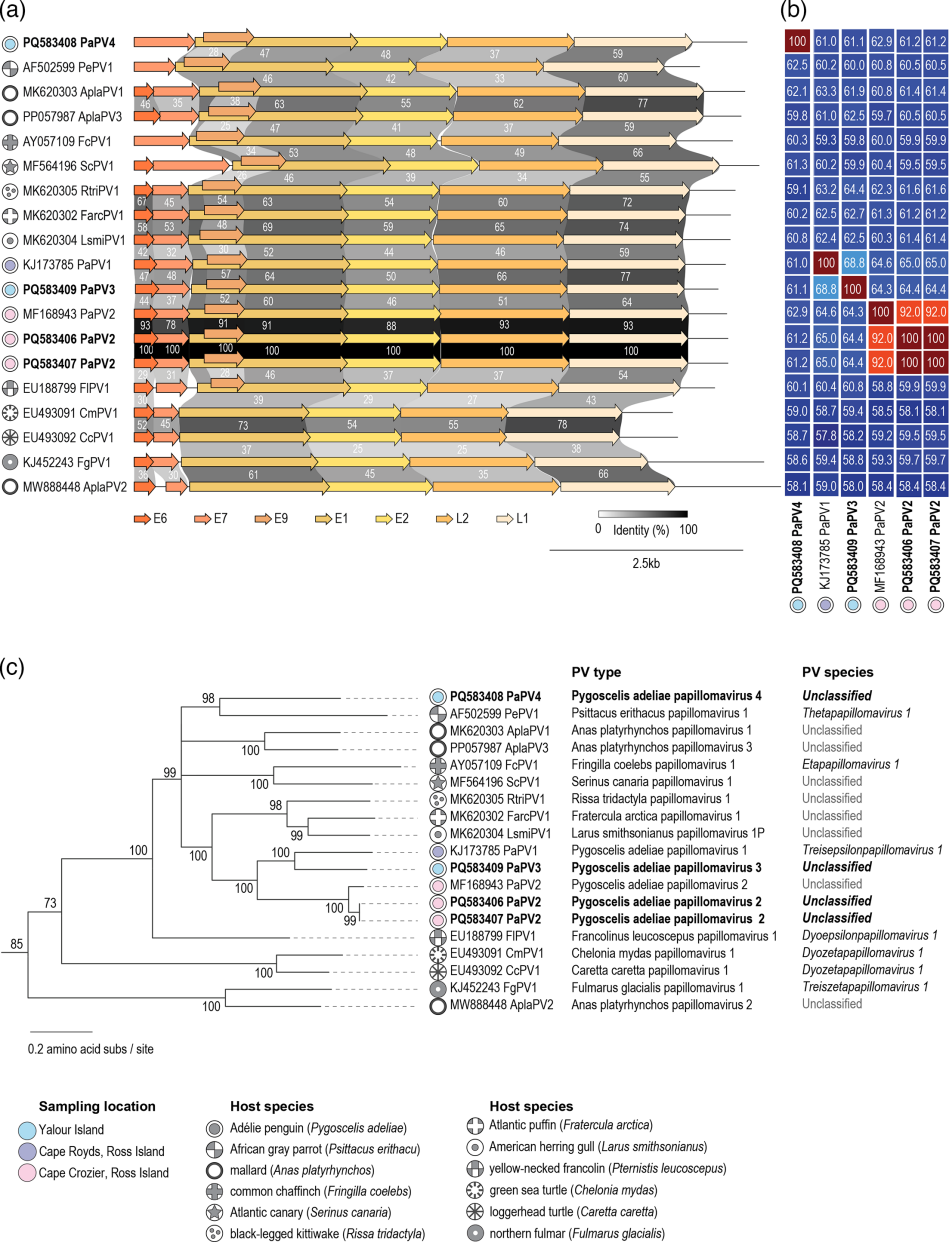
(a) Genomic organization of representative members of avian papillomaviruses and two turtle papillomaviruses. The percentage pairwise identity of the proteins E6, E7, E9, E1, E2, L1 and L2 encoded by the genomes with their nearest neighbours is shown. (b) Genome-wide pairwise identity matrix of the avian papillomaviruses and two turtle papillomaviruses. (c) Maximum-likelihood phylogenetic tree of the concatenated E1, E2 and L1 protein sequences of representative papillomaviruses. The phylogenetic tree is rooted with the gecko papillomaviruses (HfrePV1, MK207055 and HfrePV2, MN194600) [80]. Branches with <80% branch support have been collapsed using TreeGraph2 [77].

We identified four papillomaviruses, all in adult Adélie penguins, two from Cape Crozier in 2023 and two from Yalour Island in 2022 breeding season (Figs2  4, Table 2). Prior to this study, two papillomaviruses had been identified from Adélie penguins at Cape Crozier on Ross Island that represent two distinct types, i.e. *P. adeliae* papillomavirus 1 (PaPV1) [18] and *P. adeliae* papillomavirus 2 (PaPV2) [17]. Papillomavirus classification outlined by the ICTV *Papillomaviridae* study group is based on L1 gene sequence similarity with species threshold of 70% nucleotide pairwise identity and type threshold of 10% nucleotide pairwise identity [28]. The two papillomaviruses from Adélie penguins as part of this study from Cape Crozier share 92% genome-wide pairwise identity and L1 nucleotide pairwise identity of 91.7% with PaPV2 (Figs 4 and S3 in Supplementary Material). Thus, these two papillomaviruses are assigned to type PaPV2. The two from Yalour represent two different types which we have named PaPV3 and PaPV4 as they share <69% L1 gene pairwise identity with any of those other avian papillomaviruses. The four papillomavirus genomes range in size from 7,542 to 7,915 nt, with a G+C content of 45.3–53%. We did not identify any SNPs with >0.25 frequency in the reads mapped to the four papillomavirus genomes from the individual libraries. The L1 gene of the previously described PaPV1 and PaPV3 shares 71.4% nucleotide identity and thus is a member of the same species, *Treisepsilonpapillomavirus 1*. PaPV2 shares <68% and PaPV4 <64.2% pairwise L1 nucleotide identity with any other avian papillomaviruses, and thus, they represent a new species. Based on the maximum-likelihood phylogenetic tree of the concatenated E1+E2+L1 protein sequences (Fig. 4), PaPV1, PaPV2 and PaPV3 form a monophyletic clade and thus all are part of the genus *Treisepsilonpapillomavirus*. On the other hand, although PaPV4 clusters with *Psittacus erithacus* papillomavirus 1 (PePV1) in the species *Thetapapillomavirus 1*, given the pairwise identity and branch length, it may be assigned to a new genus. The four papillomaviruses have a typical genome organization found in most avian papillomaviruses (Fig. 4). PaPV4, like PePV1 and *Fringilla coelebs* papillomavirus 1, lacks an E6 gene.

PaPV2s have been identified previously from an Adélie penguin sampled at Cape Crozier during the 2014 breeding season [17], in addition to the two identified in this study from samples collected in the 2023 breeding season. At both Cape Crozier on Ross Island and Yalour Island, there are multiple PaPV types circulating in Adélie penguins, with PaPV2 having been identified in multiple individuals as well as in two independent studies sampled 9 years apart. The papillomaviruses have a relatively low incidence (1.5% for PaPV3 and PaPV4) amongst the 66 Adélie penguins sampled in western Antarctica (Antarctic Peninsula region) and 0.8% for PaPV2 amongst the 246 Adelie penguins sampled on Ross Island in eastern Antarctica. In general, compared to human papillomaviruses, those in avian species have been poorly studied with limited information on incidence rates. A study by [99] showed that the incidence of papillomaviruses varied substantially across avian species (e.g. 27.6% in *Anas* species, 9.8% in Atlantic puffin (*Fratercula arctica*), 17% in American herring gull (*Larus smithsonianus*), 2.6% in great black-backed gull (*Larus marinus*) and 81.3% in black-legged kittiwake (*Rissa tridactyla*).

All papillomaviruses have a similar genome organization encoding early (typically E6, E7, E1 and E2) and late (L1 and L2) genes. The E1 and E2 are early genes whose protein products regulate viral replication and transcription [100,103]. The E9 gene, which overlaps with the E1 coding region, is thought to repress viral gene expression [17]. It is found in most avian papillomaviruses, with the exception of *Fulmarus glacialis* papillomavirus 1 from northern fulmar (*F. glacialis*) [104] and *Anas platyrhynchos* papillomavirus 2 from mallard (*A. platyrhynchos*) [105]. The E6 and E7 genes encode oncoproteins and are key to papillomavirus pathogenesis [106,107].

While mammalian PVs typically have two zinc-binding motifs within the E6 protein, avian papillomaviruses contain a single zinc-binding motif [108]. A comparative analysis of the E6 protein encoded by the three penguin papillomavirus types shows this single zinc-binding motif, which is common in avian and two turtle papillomaviruses (Fig. S4 in Supplementary Material). E7 protein of papillomaviruses has a conserved LxCxE binding motif, which facilitates interaction and binding of the tumour suppressor gene retinoblastoma (pRB) [109]. In addition to the LxCxE binding motif, E7 is a metalloprotein that, like E6, mediates zinc binding through cysteine residues. These binding motifs are responsible for the oncogenicity in the protein [110]. In the E7 of all penguin papillomaviruses, we identified one Cx2Cx-23-Cx2C canonical zinc-binding motif and an LxCxE motif (Fig. S4 in Supplementary Material) Furthermore, in all the penguin papillomaviruses, we identified the E9 gene that is found in most avian papillomaviruses (Fig. 4). This protein product of this gene is not homologous to other known proteins and thus its function remains unknown. Pairwise identity comparisons of the E9 protein show it exhibits high diversity amongst the avian papillomaviruses (18–39%, Fig. S3 - Supplementary Material) The pairwise identity matrices of the E9, E7, E6, E1, L1 and L2 are provided in Figs S3 and S4 in Supplementary Material .

## Conclusion

We report 31 genomes of polyomaviruses representing four lineages from three penguin species (Adélie, chinstrap and gentoo) and four genomes of papillomaviruses representing three types from Adélie penguins. These genome sequences substantially increase what was previously known about a polyomavirus and two papillomaviruses associated with Adélie penguins on Ross Island [17,18, 22]. More broadly, it highlights the lack of knowledge regarding viruses that infect penguins, and these 35 genomes add to the small number of complete (*n*=18) and near-complete virus (*n*=26) genomes in public databases. Our finding highlights the broader distribution of polyomaviruses and papillomaviruses coupled with host and location-specific presence/detection. The identification of the diverse papillomaviruses representing at least three viral species in Adélie penguins suggests that there are likely more diverse lineages circulating amongst penguins but at a relatively low incidence (0.8–2%). In contrast, we note that although we have much higher but variable incidence rates for polyomaviruses, both across sampling sites and penguin host species, we have a substantially higher incidence of both the penguin polyomavirus 4 and penguin polyomavirus 5 lineages, and both of these are circulating amongst gentoo penguins in the Antarctic Peninsula region: Cierva Cove and Hope Bay in Trinity Peninsula and Hannah Point on Livingston Island and Stranger Point on King George Island in the South Shetland Islands. At Hannah Point on Livingston Island, there were two individual cases of co-occurrence with these two variants in gentoo penguins. This begs the question of whether co-occurrence with lineage variants of polyomaviruses is common in other avian, mammalian and fish hosts.

Our findings improve understanding of the evolutionary dynamics of these viruses with four papillomavirus types representing three viral species circulating in Adélie penguins. Moreover, it is likely that there was some level of host adaptation with geographical isolation within species and limited transmission across large geographical distances in terms of colony location. In our dataset, we only observed one case of penguin polyomavirus 2 in an individual from the Antarctic Peninsula (western Antarctica), while it was commonly associated with Adélie penguins on Ross Island in eastern Antarctica. This raises questions about whether this is a case of transmission occurring during the Austral winter period where the Adélie penguins that breed at two geographically distant locations may be interacting during winter on pack ice, or whether this is a case of dispersal [111,112] of an infected individual from the Ross Sea to the Antarctic Peninsula region. On the other hand, 10–15 Adélie penguin colonies lie between the Ross Sea and the northern Antarctic Peninsula [113,115] where we sampled, including in Marie Byrd Land, from which some Ross Island penguins spend portions of the winter [113,116]. Certainly, these populations are likely to intermingle during winter. To address this, continued surveillance and viral genomic analysis within a broader geographical framework is needed, coupled with data on movement of penguins in the non-breeding periods, in particular austral winters when they are dispersing into the pack ice area.

## Supplementary material

10.1099/mgen.0.001580 Supplementary Material 1.

## Abbreviations

HfrePV1: *Hemidactylus frenatus* papillomavirus 1
HfrePV2: *Hemidactylus frenatus* papillomavirus 2
ICTV: International Committee on Taxonomy of Viruses
NCBI: National Center for Biotechnology Information
PaPV1: *Pygoscelis adeliae* papillomavirus 1
PaPV2: *Pygoscelis adeliae* papillomavirus 2
PePV1: *Psittacus erithacus* papillomavirus 1
RCA: rolling circle amplification
UTM: Universal Transport Medium
