# Supplementary material for: Diversity of polyomaviruses and papillomaviruses in penguins from eastern and western Antarctica
Source: Microb Genom. 2025 Nov 24;11(11):001580. doi: 10.1099/mgen.0.001580 (PMC12643040; doi:10.1099/mgen.0.001580)
Supplement: Supplementary Material 1. [file mgen-11-01580-s001.pdf]

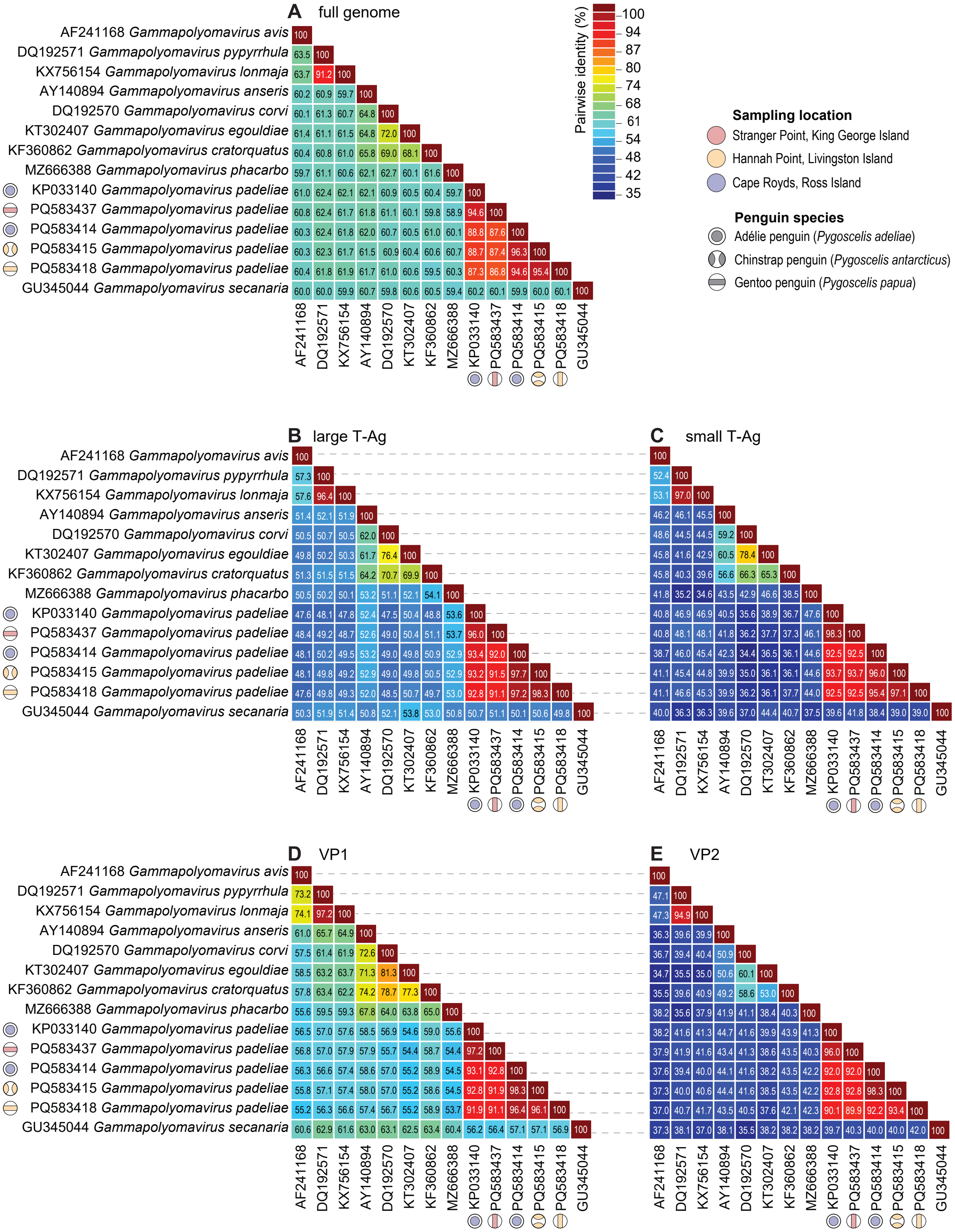

Supplementary Figure 1: Pairwise identity matrices of members of the Gammapolyomavirus genus including the five penguin polyomavirus lineages identified in Adélie, chinstrap, and gentoo penguins. **A**. Genome-wide pairwise nucleotide identity. **B**. Pairwise identity of the large T-antigen protein sequences. **C**. Pairwise identity of the small T-antigen protein sequences. **D**. Pairwise identity of the VP1 protein sequences. **E**. Pairwise identity of the VP2 protein sequences.

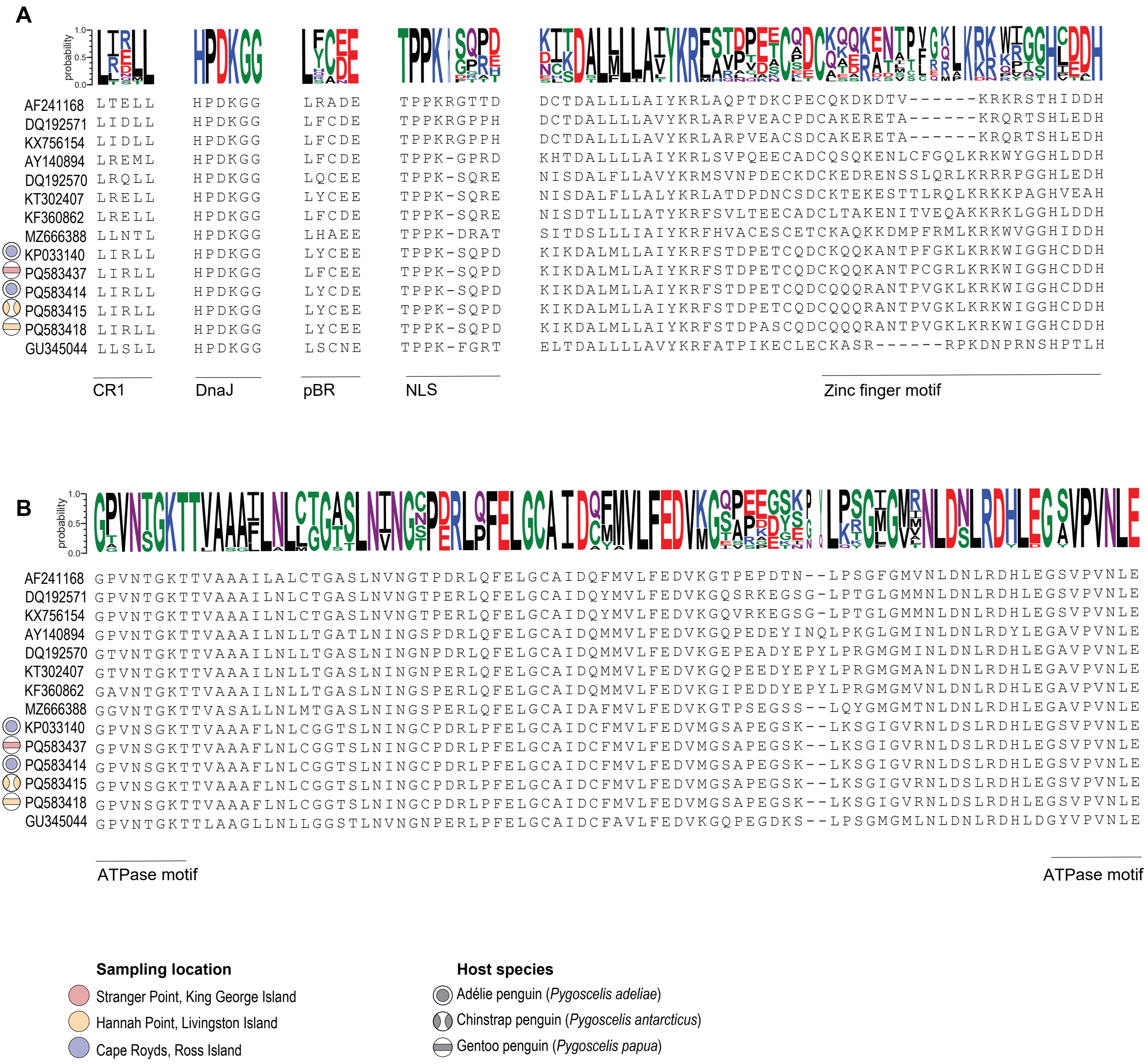

Supplementary Figure 2: Sequence logos of the conserved motifs identified in the large T-antigen encoded by members of the Gammapolyomavirus genus, including those of the five lineages associated penguins. A. The conserved motifs of conserved region 1 (CR1), Hexapeptide (DnaJ), Retinoblastoma protein binding (pRB), Putative nuclear localization signal (NLS), Zinc finger motif. **B**. The conserved ATPase motifs.

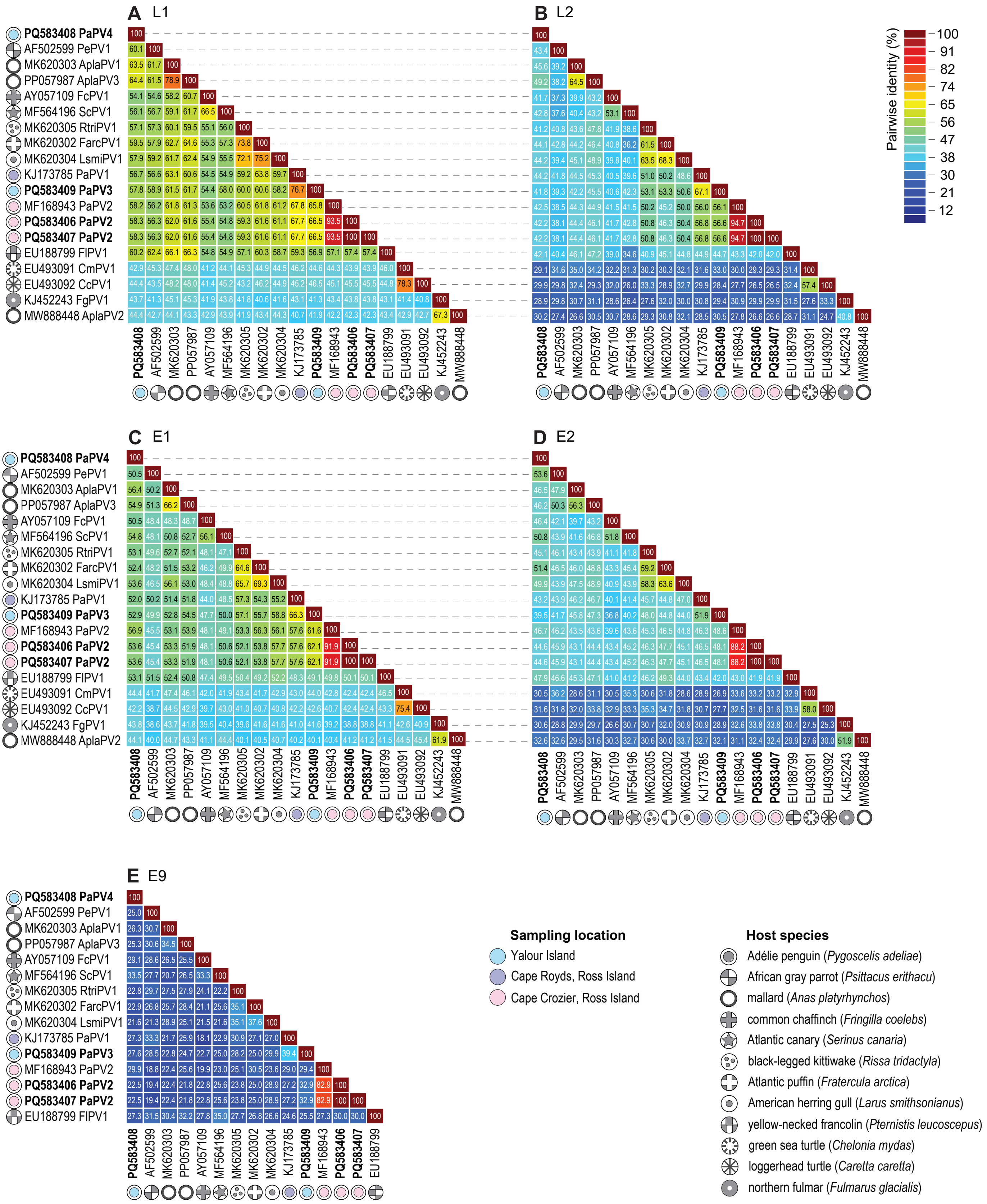

Supplementary Figure 3. Pairwise identity matrices of L1, L2, E1, E2, and E9 proteins encoded by representative members of avian papillomaviruses and two turtle ones. **A**. Pairwise identity of the L1 protein sequences. **B**. Pairwise identity of the L2 protein sequences. **C**. Pairwise identity of the E1 protein sequences. **D**. Pairwise identity of the E2 protein sequences. E. Pairwise identity of the E9 protein sequences.

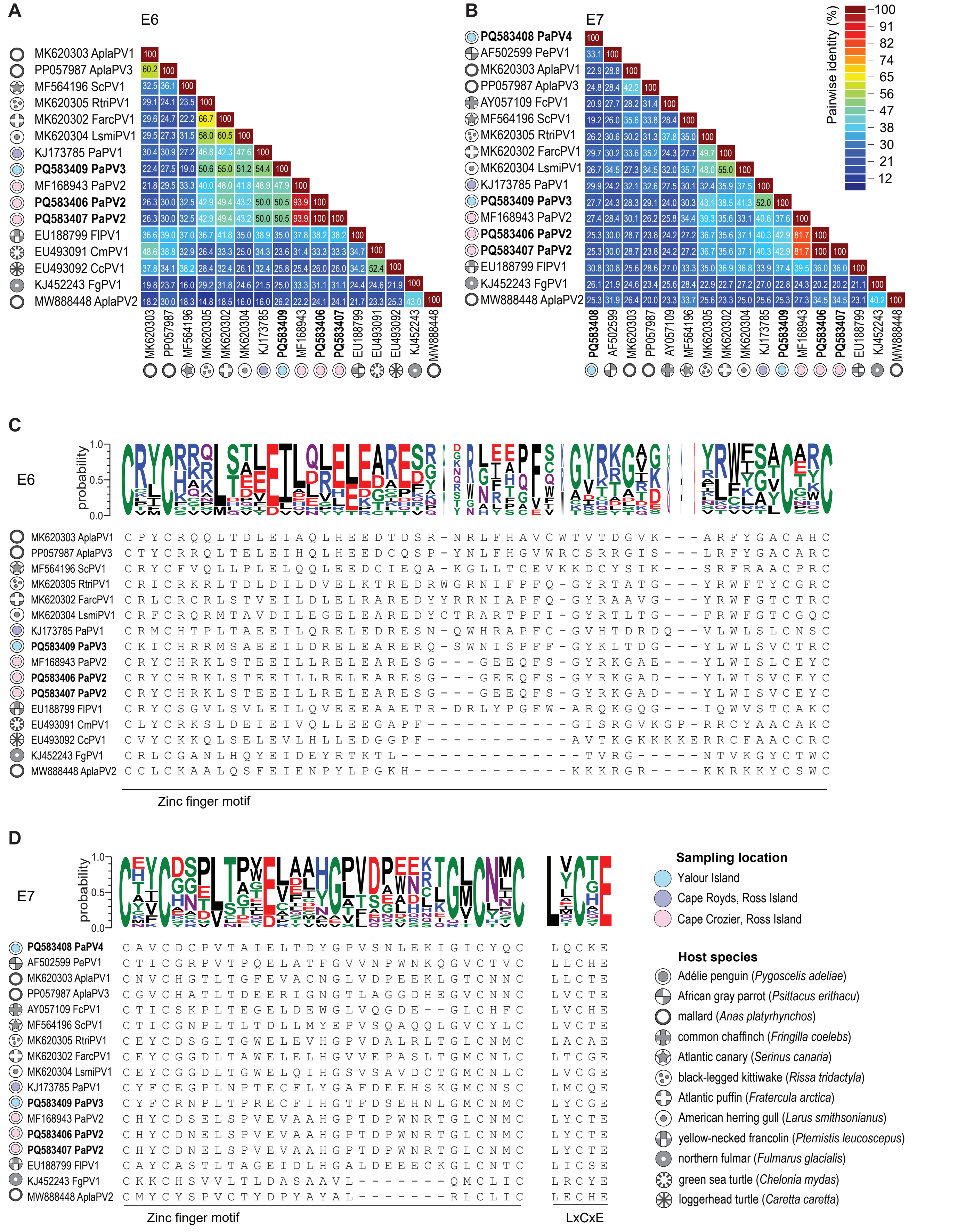

Supplementary Figure 4: Protein sequence analysis of the E6 and E7 proteins. **A**. Pairwise identity of the E6 protein sequences. **B**. Pairwise identity of the E9 protein sequences. **C**. Sequence logos of the zinc finger motif present in the E6 protein sequences. **D**. Sequence logos of the zinc finger and LxCxE motifs in the E7 protein sequences.
